# Supplementary material for: Pyruvate Dehydrogenase Kinase 1 inhibition mediated oxidative phosphorylation enhancement in cartilage promotes osteoarthritis progression
Source: BMC Musculoskelet Disord. 2023 Jul 20;24:597. doi: 10.1186/s12891-023-06585-6 (PMC10357736; doi:10.1186/s12891-023-06585-6)
Supplement: Supplementary file 1 — Additional file 1. [file 12891_2023_6585_MOESM1_ESM.zip › KEGG.Gsea.1653622432464/KEGG_STARCH_AND_SUCROSE_METABOLISM.html]

Details for gene set KEGG\_STARCH\_AND\_SUCROSE\_METABOLISM[GSEA]

|  || Dataset | 55457\_collapsed\_to\_symbols.cls.cls #OA\_versus\_normal.cls.cls #OA\_versus\_normal\_repos |
| Phenotype | cls.cls#OA\_versus\_normal\_repos |
| Upregulated in class | OA |
| GeneSet | KEGG\_STARCH\_AND\_SUCROSE\_METABOLISM |
| Enrichment Score (ES) | 0.28599524 |
| Normalized Enrichment Score (NES) | 1.0777744 |
| Nominal p-value | 0.33333334 |
| FDR q-value | 0.49886838 |
| FWER p-Value | 1.0 |
Table: GSEA Results Summary

  

Fig 1: Enrichment plot: KEGG\_STARCH\_AND\_SUCROSE\_METABOLISM      
 Profile of the Running ES Score & Positions of GeneSet Members on the Rank Ordered List

  

| SYMBOL | TITLE | RANK IN GENE LIST | RANK METRIC SCORE | RUNNING ES | CORE ENRICHMENT || 1 | UXS1 | UDP-glucuronate decarboxylase 1 [Source:HGNC Symbol;Acc:HGNC:17729] | 91 | 0.926 | 0.0792 | Yes |
| 2 | GPI | glucose-6-phosphate isomerase [Source:HGNC Symbol;Acc:HGNC:4458] | 166 | 0.842 | 0.1518 | Yes |
| 3 | UGT2B4 | UDP glucuronosyltransferase family 2 member B4 [Source:HGNC Symbol;Acc:HGNC:12553] | 566 | 0.634 | 0.1798 | Yes |
| 4 | GUSB | glucuronidase beta [Source:HGNC Symbol;Acc:HGNC:4696] | 904 | 0.541 | 0.2040 | Yes |
| 5 | GAA | alpha glucosidase [Source:HGNC Symbol;Acc:HGNC:4065] | 1149 | 0.486 | 0.2303 | Yes |
| 6 | GBA3 | glucosylceramidase beta 3 (gene/pseudogene) [Source:HGNC Symbol;Acc:HGNC:19069] | 1767 | 0.388 | 0.2185 | Yes |
| 7 | PYGM | "glycogen phosphorylase, muscle associated [Source:HGNC Symbol;Acc:HGNC:9726]" | 1952 | 0.367 | 0.2384 | Yes |
| 8 | UGT2B17 | UDP glucuronosyltransferase family 2 member B17 [Source:HGNC Symbol;Acc:HGNC:12547] | 2050 | 0.356 | 0.2640 | Yes |
| 9 | PYGB | glycogen phosphorylase B [Source:HGNC Symbol;Acc:HGNC:9723] | 2400 | 0.315 | 0.2662 | Yes |
| 10 | UGT2A3 | UDP glucuronosyltransferase family 2 member A3 [Source:HGNC Symbol;Acc:HGNC:28528] | 2854 | 0.265 | 0.2556 | Yes |
| 11 | UGT1A8 | UDP glucuronosyltransferase family 1 member A8 [Source:HGNC Symbol;Acc:HGNC:12540] | 3301 | 0.221 | 0.2415 | Yes |
| 12 | ENPP1 | ectonucleotide pyrophosphatase/phosphodiesterase 1 [Source:HGNC Symbol;Acc:HGNC:3356] | 3392 | 0.212 | 0.2543 | Yes |
| 13 | AGL | "amylo-alpha-1, 6-glucosidase, 4-alpha-glucanotransferase [Source:HGNC Symbol;Acc:HGNC:321]" | 3537 | 0.199 | 0.2616 | Yes |
| 14 | HK3 | hexokinase 3 [Source:HGNC Symbol;Acc:HGNC:4925] | 3792 | 0.178 | 0.2584 | Yes |
| 15 | PYGL | glycogen phosphorylase L [Source:HGNC Symbol;Acc:HGNC:9725] | 3826 | 0.174 | 0.2720 | Yes |
| 16 | PGM1 | phosphoglucomutase 1 [Source:HGNC Symbol;Acc:HGNC:8905] | 3990 | 0.157 | 0.2740 | Yes |
| 17 | SI | sucrase-isomaltase [Source:HGNC Symbol;Acc:HGNC:10856] | 4021 | 0.154 | 0.2860 | Yes |
| 18 | UGT1A10 | UDP glucuronosyltransferase family 1 member A10 [Source:HGNC Symbol;Acc:HGNC:12531] | 4850 | 0.083 | 0.2294 | No |
| 19 | G6PC1 | glucose-6-phosphatase catalytic subunit 1 [Source:HGNC Symbol;Acc:HGNC:4056] | 5389 | 0.041 | 0.1914 | No |
| 20 | GYS2 | glycogen synthase 2 [Source:HGNC Symbol;Acc:HGNC:4707] | 6316 | -0.029 | 0.1221 | No |
| 21 | HK1 | hexokinase 1 [Source:HGNC Symbol;Acc:HGNC:4922] | 6728 | -0.058 | 0.0956 | No |
| 22 | UGT2B11 | UDP glucuronosyltransferase family 2 member B11 [Source:HGNC Symbol;Acc:HGNC:12545] | 7270 | -0.100 | 0.0629 | No |
| 23 | GYS1 | glycogen synthase 1 [Source:HGNC Symbol;Acc:HGNC:4706] | 7345 | -0.105 | 0.0669 | No |
| 24 | UGP2 | UDP-glucose pyrophosphorylase 2 [Source:HGNC Symbol;Acc:HGNC:12527] | 8735 | -0.211 | -0.0213 | No |
| 25 | G6PC2 | glucose-6-phosphatase catalytic subunit 2 [Source:HGNC Symbol;Acc:HGNC:28906] | 9088 | -0.239 | -0.0265 | No |
| 26 | UGT2A1 | UDP glucuronosyltransferase family 2 member A1 complex locus [Source:HGNC Symbol;Acc:HGNC:12542] | 9451 | -0.269 | -0.0295 | No |
| 27 | TREH | trehalase [Source:HGNC Symbol;Acc:HGNC:12266] | 9910 | -0.313 | -0.0360 | No |
| 28 | HK2 | hexokinase 2 [Source:HGNC Symbol;Acc:HGNC:4923] | 10030 | -0.325 | -0.0151 | No |
| 29 | MGAM | maltase-glucoamylase [Source:HGNC Symbol;Acc:HGNC:7043] | 10106 | -0.331 | 0.0100 | No |
| 30 | GCK | glucokinase [Source:HGNC Symbol;Acc:HGNC:4195] | 10137 | -0.335 | 0.0388 | No |
| 31 | UGDH | UDP-glucose 6-dehydrogenase [Source:HGNC Symbol;Acc:HGNC:12525] | 10279 | -0.349 | 0.0603 | No |
| 32 | GBE1 | "1,4-alpha-glucan branching enzyme 1 [Source:HGNC Symbol;Acc:HGNC:4180]" | 10356 | -0.357 | 0.0876 | No |
| 33 | AMY1A | amylase alpha 1A [Source:HGNC Symbol;Acc:HGNC:474] | 11681 | -0.529 | 0.0339 | No |
| 34 | UGT2B15 | UDP glucuronosyltransferase family 2 member B15 [Source:HGNC Symbol;Acc:HGNC:12546] | 12225 | -0.652 | 0.0525 | No |
Table: GSEA details [plain text format]

  

Fig 2: KEGG\_STARCH\_AND\_SUCROSE\_METABOLISM      
 Blue-Pink O' Gram in the Space of the Analyzed GeneSet

  

Fig 3: KEGG\_STARCH\_AND\_SUCROSE\_METABOLISM: Random ES distribution      
 Gene set null distribution of ES for **KEGG\_STARCH\_AND\_SUCROSE\_METABOLISM**
